# Supplementary material for: A tuneable minimal cell membrane reveals that two lipid species suffice for life
Source: Nat Commun. 2024 Nov 8;15:9679. doi: 10.1038/s41467-024-53975-y (PMC11549477; doi:10.1038/s41467-024-53975-y)
Supplement: Supplementary file 1 — Supplementary Information [file 41467_2024_53975_MOESM1_ESM.pdf]

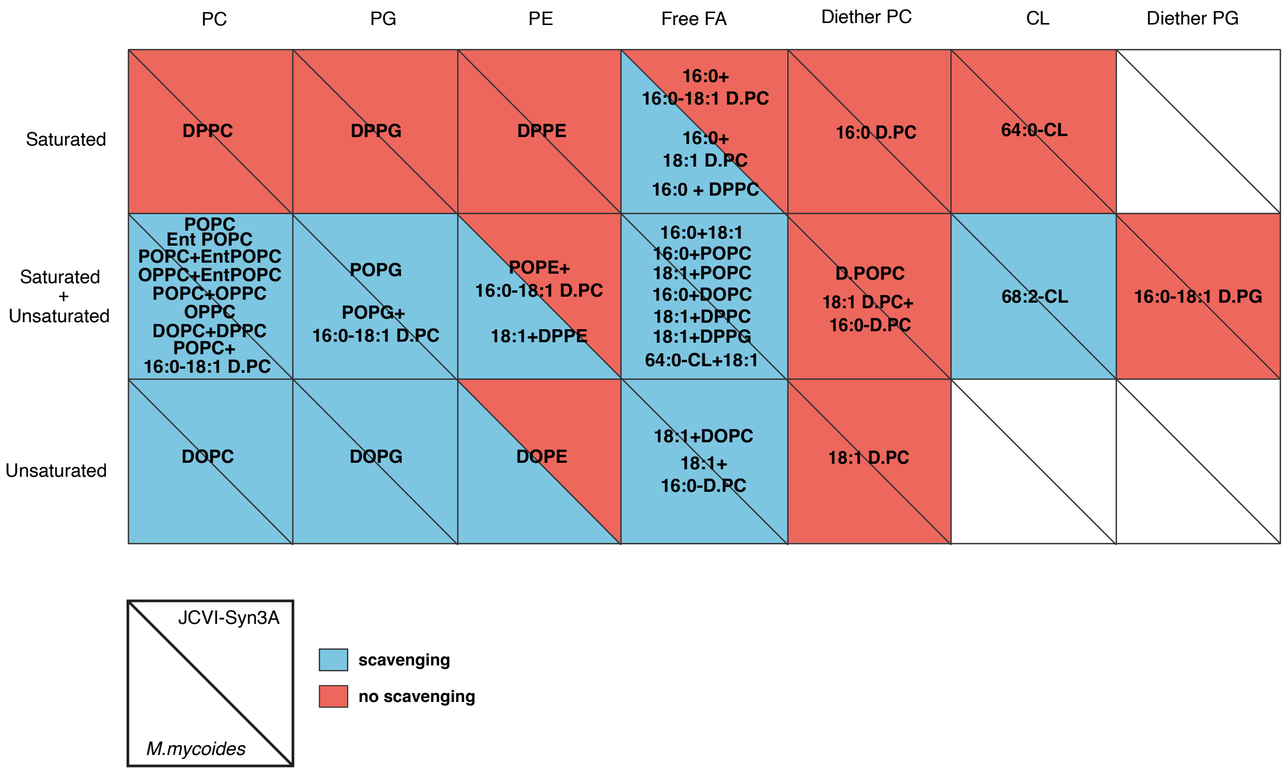

<u>Figure S1: Certain Lipid Features Determine Cells' Ability to Scavenge Acyl Chains</u>

All of the lipid diets fed to *M. mycoides* and JCVI-Syn3A were sorted based on lipid features, specifically by head group and acyl chain saturation, and the ability of *M. mycoides* and JCVI-Syn3A to scavenge acyl chains was determined by TLC analysis (Figure S4), with production of PG and Cardiolipin being taken as proof of scavenging occurring. As a result of this sorting, certain lipid features that lead to scavenging capacity, or the lack thereof, could be looked at. When only saturated phospholipids are fed, neither *M. mycoides* nor JCVI-Syn3A can scavenge acyl chains. When free 16:0 is added to the diet *M. mycoides* is able to produce PG and Cardiolipin, while JCVI-Syn3A is not. Furthermore, JCVI-Syn3A is unable to scavenge acyl chains from PE species of lipid, regardless of saturation.

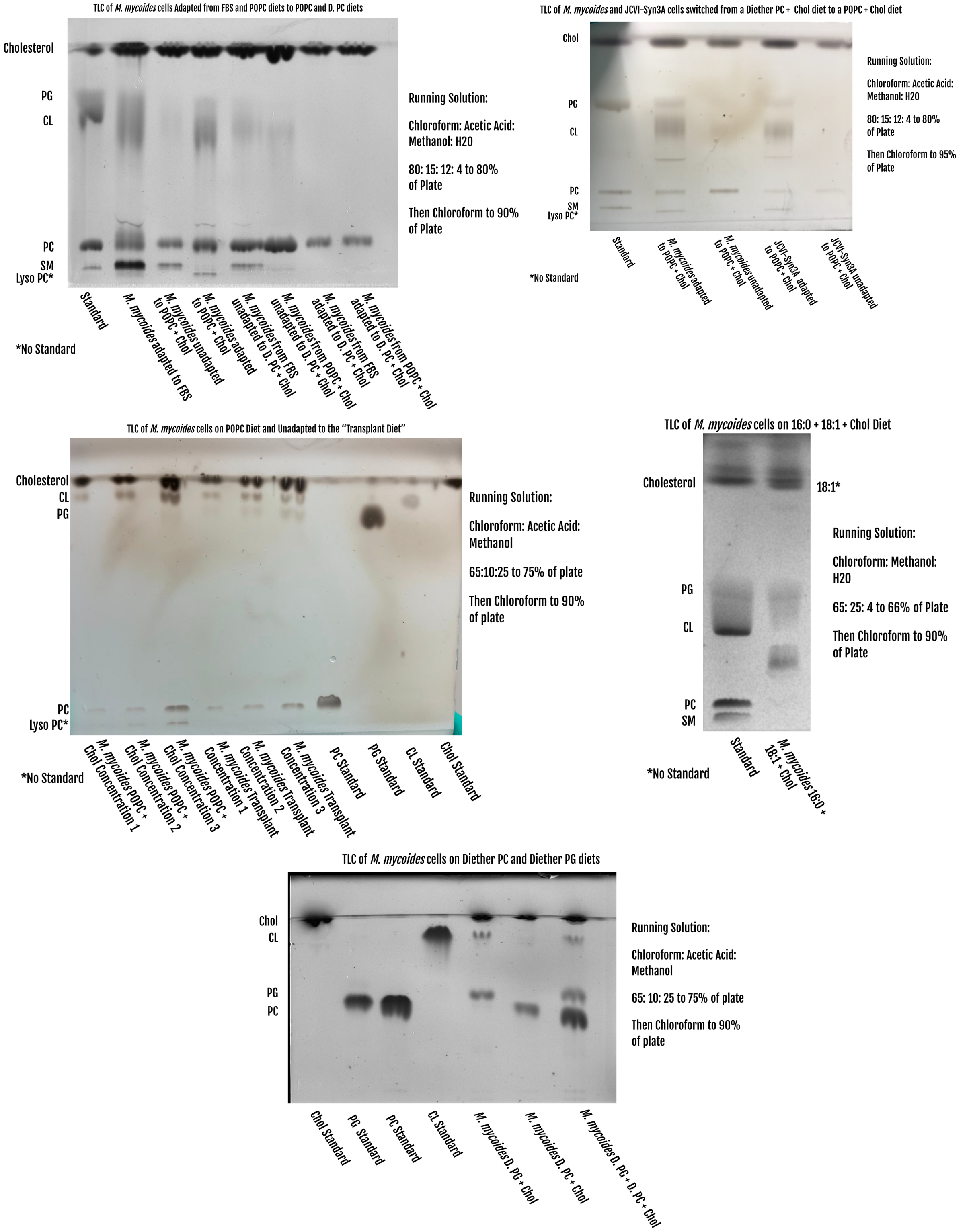

<u>Figure S2: TLC Analysis of Lipid Classes Present in Whole Cell Lipid Extracts</u>

The synthesis of PG and Cardiolipin by *M. mycoides* and JCVI-Syn3A fed on diets lacking free fatty acids provides evidence of acyl chain scavenging, as fatty acids are required to synthesize these lipid classes. To assay PG and Cardiolipin production, whole cell lipid extractions were performed on both organisms, which were then separated and visualized using thin layer chromatography against lipid standards to determine the presence or absence of PG and Cardiolipin. All plates are annotated.

S3a

S3b

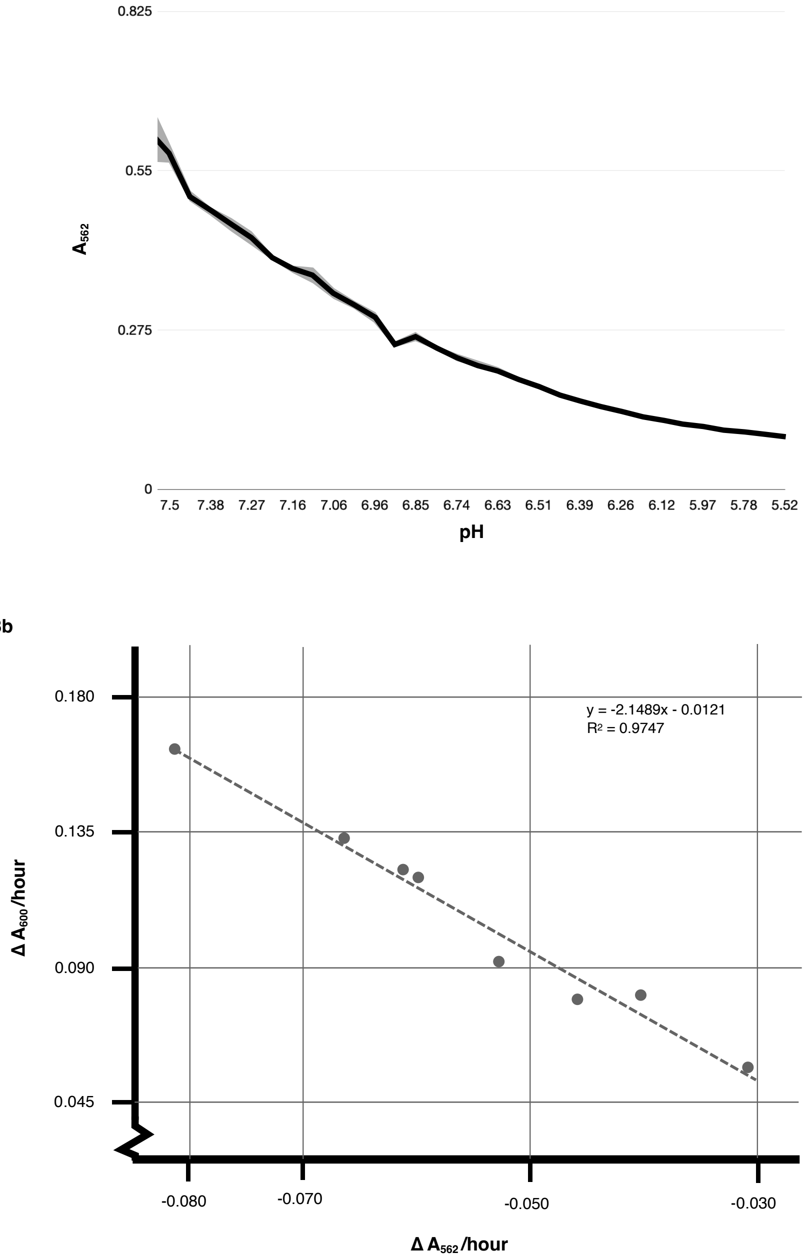

Figure S3: Validating Phenol Red Absorbance Rates as a Method to Measure Cell Growth

a The absorbance of phenol red in SP4 media at 562nm (A_{562}) as a function pH measured with an acid titration shows how phenol red signal A_{562} decreases as media is acidified. **b** A scatterplot showing the relationship between growth rate values for *M. mycoides* obtained by measuring the optical density of cells at 600nm (OD_{600}) and metabolic growth index rates obtained by measuring changes in pH by looking at phenol red A_{562}. Eight diets were chosen to span a range of lipid species, saturations, and inclusions or exclusions of free fatty acids to ensure that they were representative of all diets. Measuring growth with phenol red A_{562} was chosen due to difficulties obtaining reproducible OD_{600} growth rates for JCVI-Syn3A. Furthermore, differences in cell size and growth behavior lead to difficulties properly comparing *M. mycoides* and JCVI-Syn3A values obtained from OD_{600}. Growth rates obtained by OD_{600} are consistently ~2-fold higher than those obtained from phenol red A_{562}

S4a

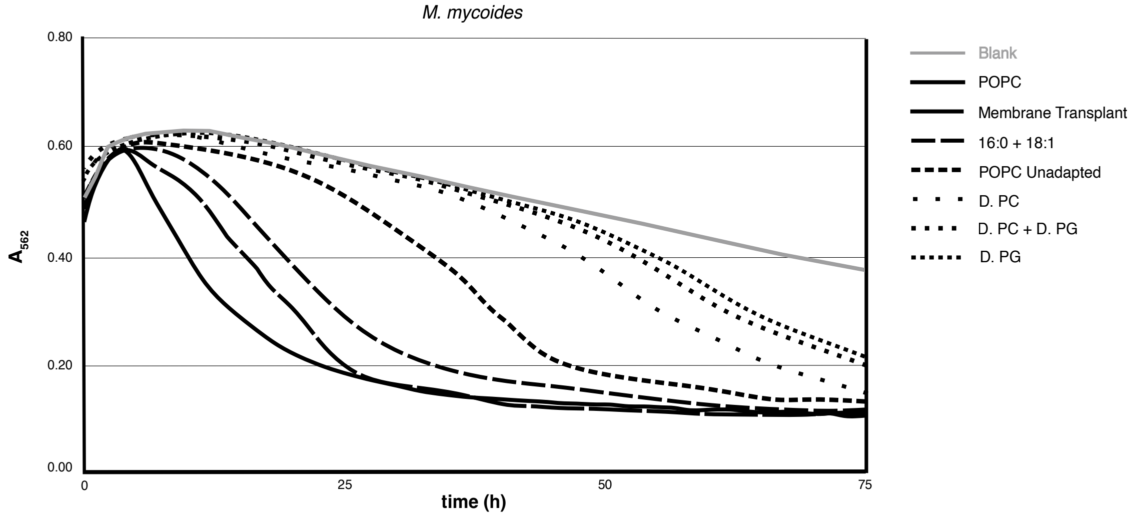

S4b

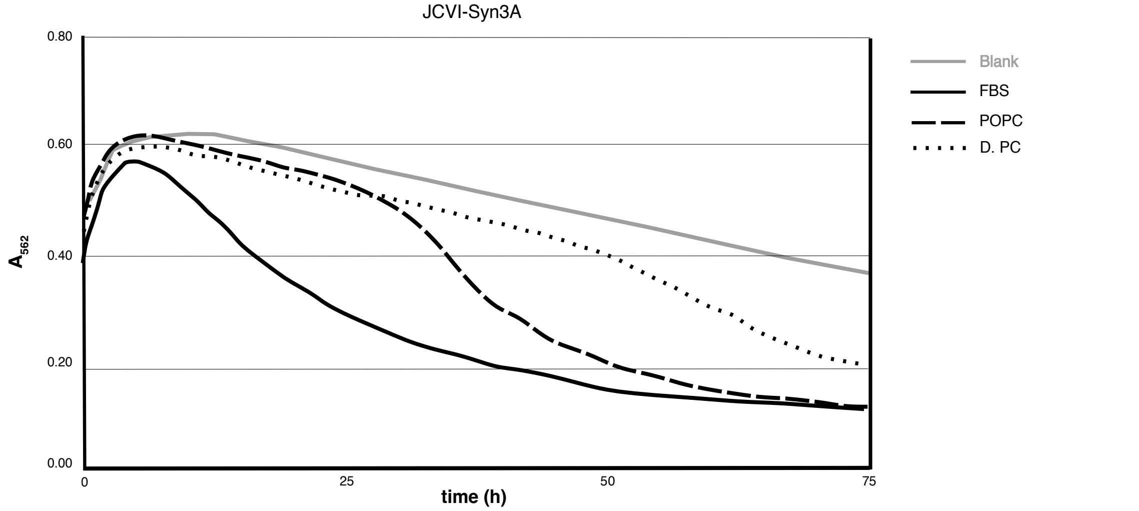

S4c

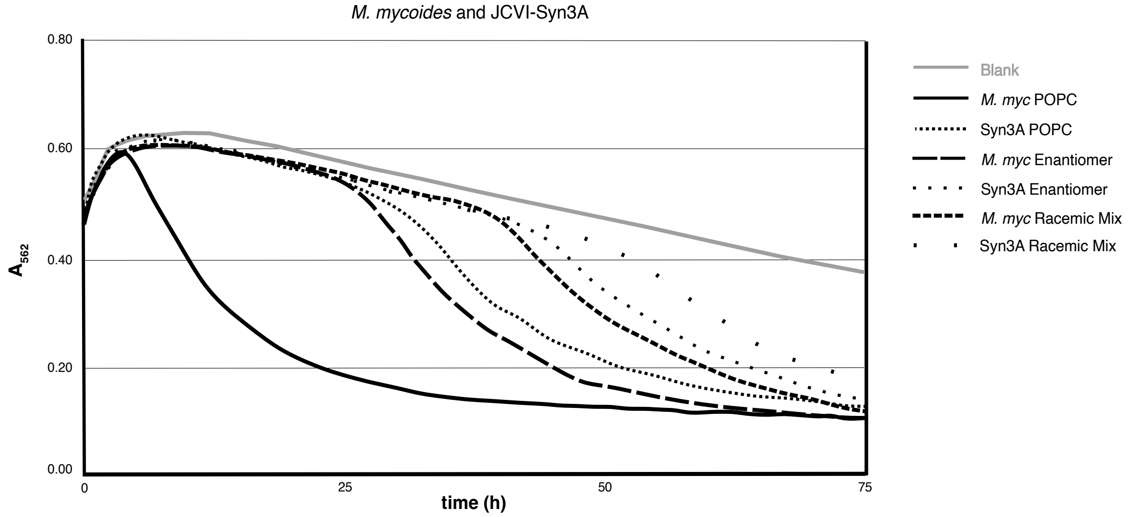

<u>Figure S4: Full A_{562} Growth Curves</u>

A plot of the full average A_{562} growth curves for all conditions in **a** Figure 3, **b** Figure 4, and **c** Figure 5. Growth rates were calculated by taking an exponential fit of the most exponential part of each growth curve.

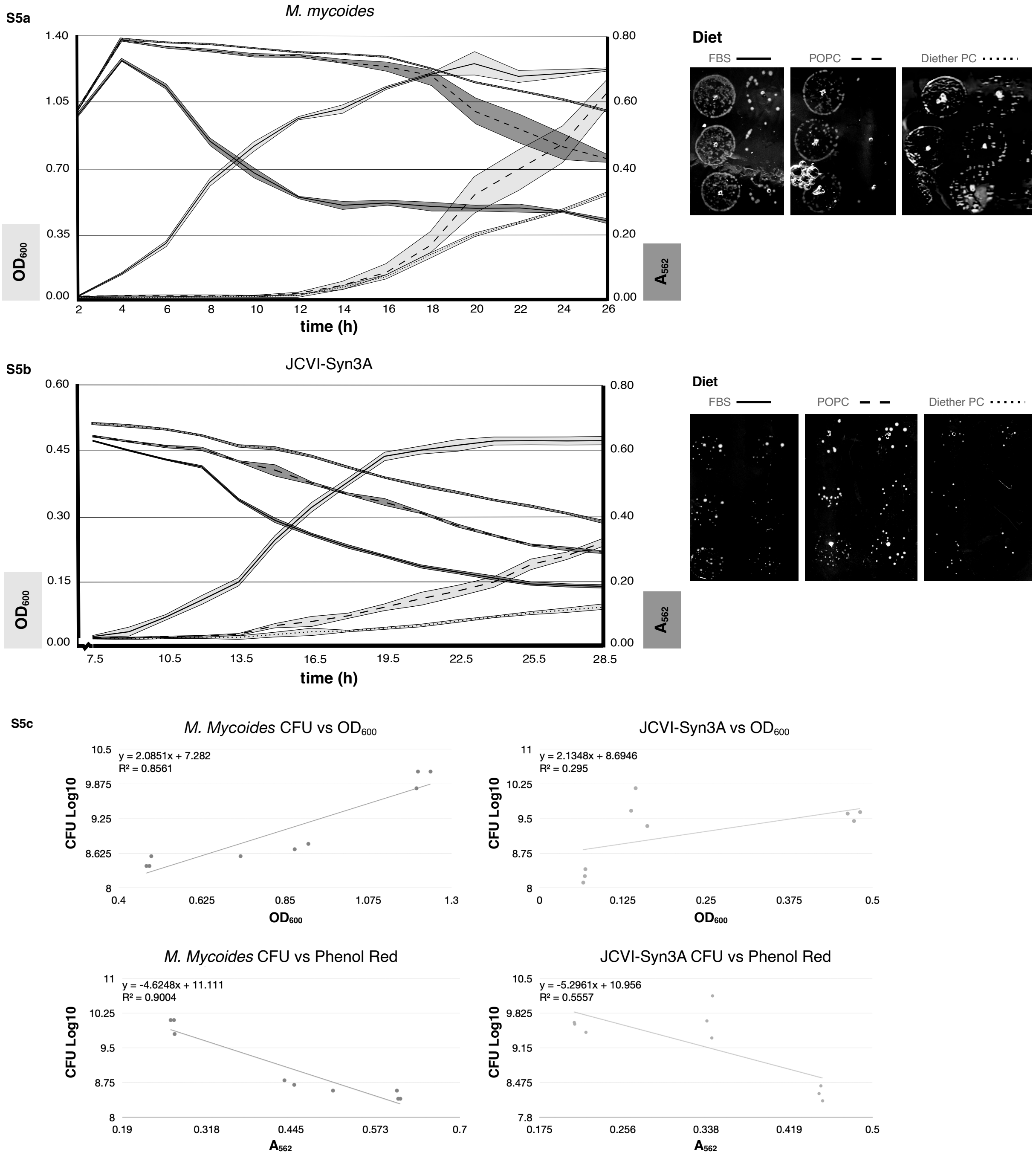

Figure S5: Growth of *M. mycoides* and JCVI-Syn3A manually measured by OD_{600}, phenol red A_{562}, and Colony-Forming Units

a Growth curves for *M. mycoides* on FBS, POPC, and Diether PC diets in phenol red media obtained by manual measurements of OD_{600} and A_{562}. At 24 hours, cells were taken for each condition and serially diluted on a SP4+FBS plate to obtain a count of colony-forming units.
b Growth curves for JCVI-Syn3A cells on FBS, POPC, and Diether PC diets in phenol red media obtained by manual measurements of OD_{600} and A_{562}. At 24 hours, cells were taken for each condition and serially diluted on a SP4+FBS plate to obtain a count of colony-forming units. **c** Comparisons between CFUs, OD_{600} and A_{562} readings show a correlation between higher OD_{600} and A_{562} values and more CFUs, validating the absorbance-based methods of measuring growth. The correlation was weakest for JCVI-Syn3A cells measured by OD_{600}, further validating the decision to use phenol red A_{562}-based growth assays.

S6a

FBS overview

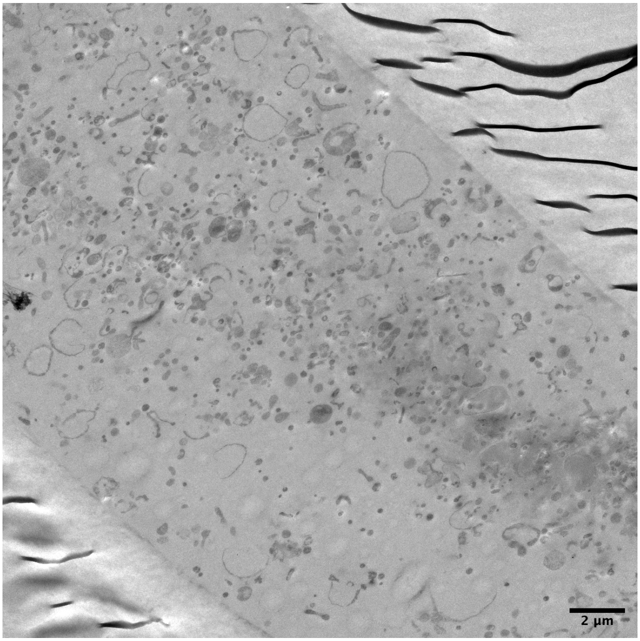

FBS overview
with cell count

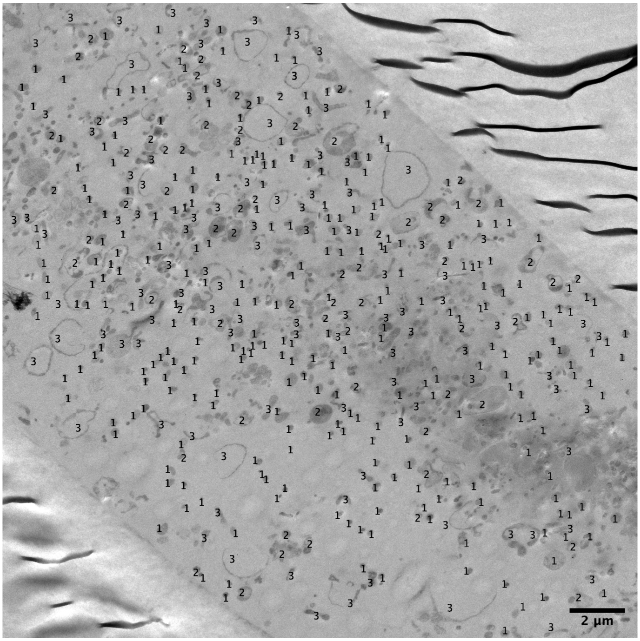

S6b

POPC overview 1

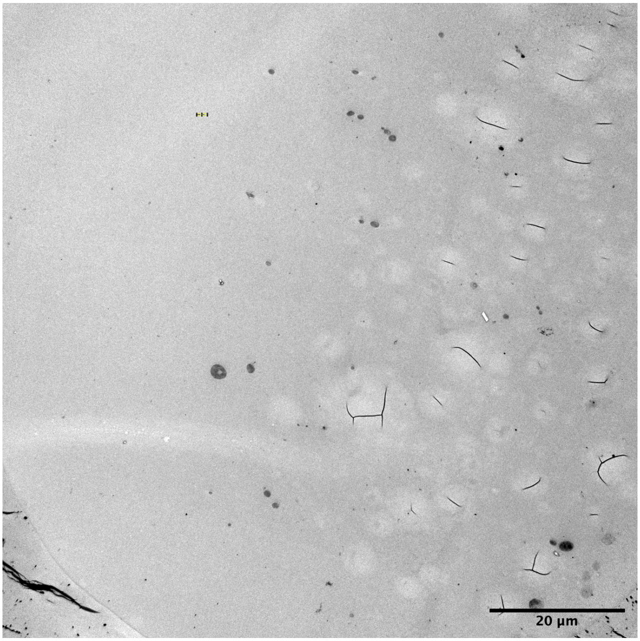

POPC overview 1 with cell count

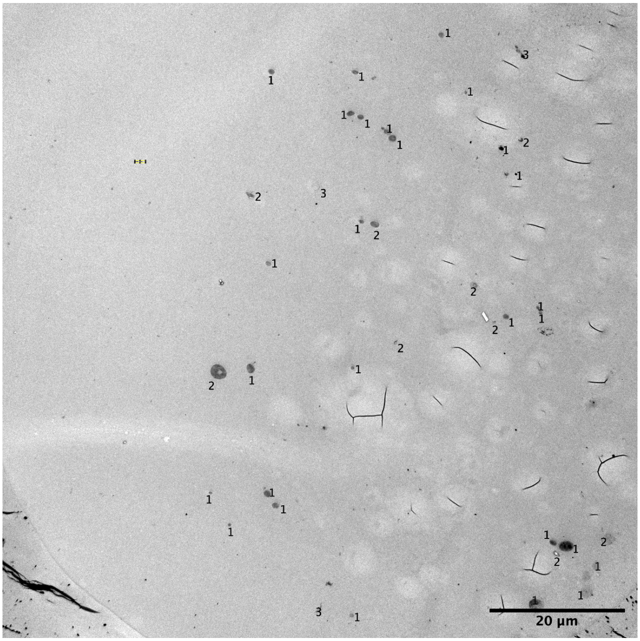

S6c

POPC overview 2

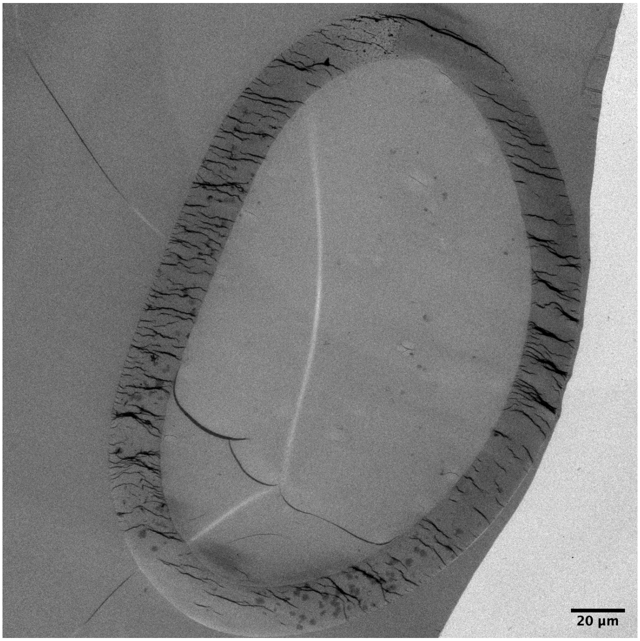

POPC overview 2 with cell count

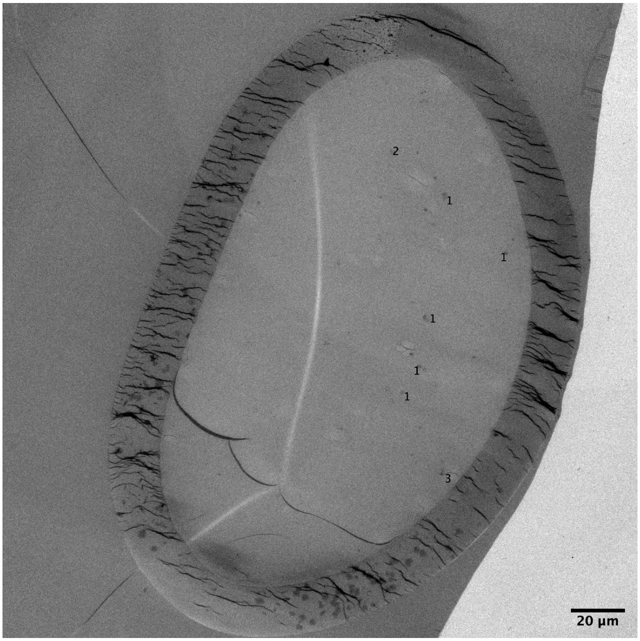

S6d

POPC overview 3

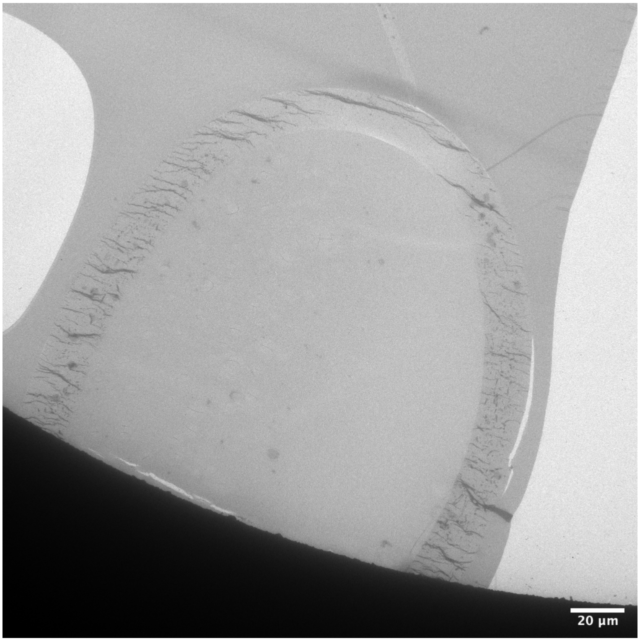

POPC overview 3 with cell count

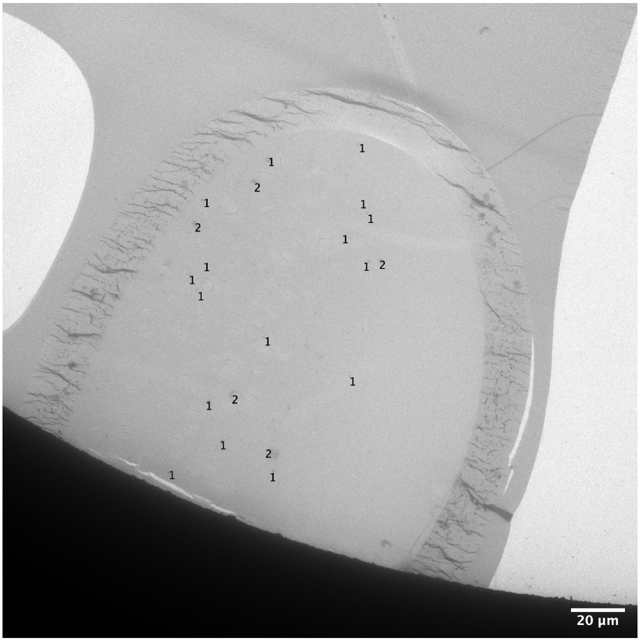

S6e

D.PC overview 1

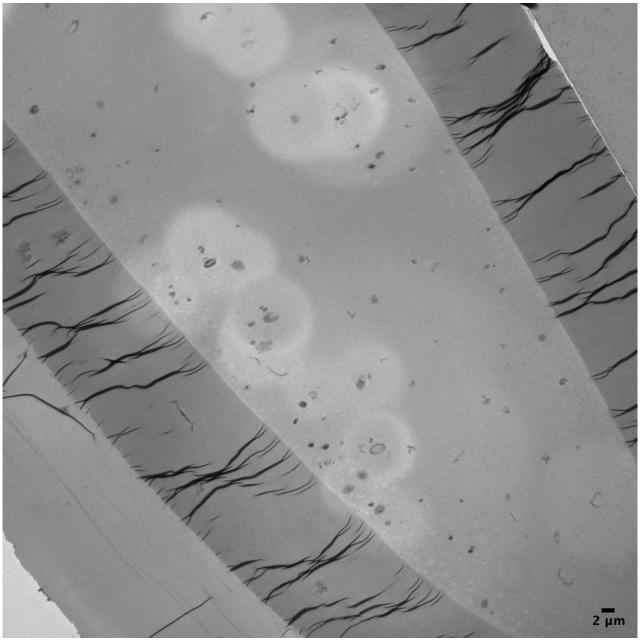

D.PC overview 1 with cell count

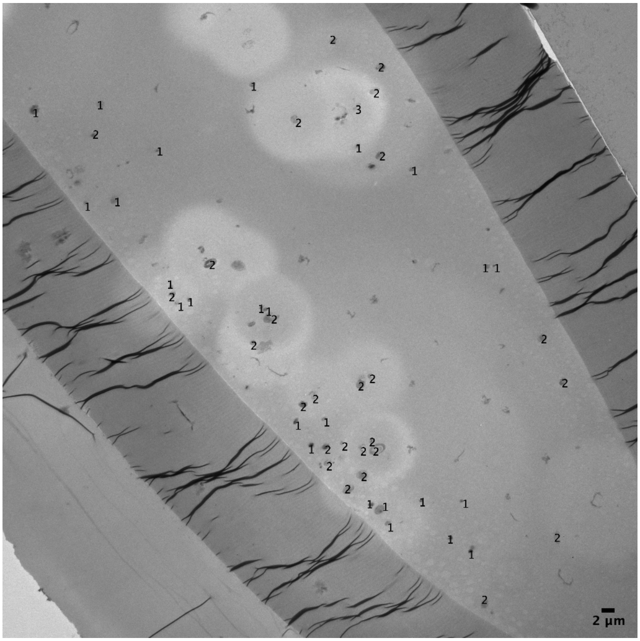

S6f

D.PC overview 2

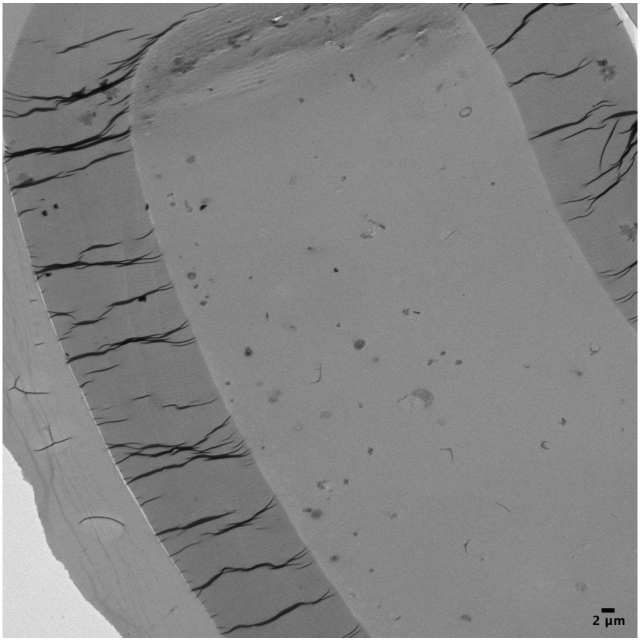

D.PC overview 2 with cell count

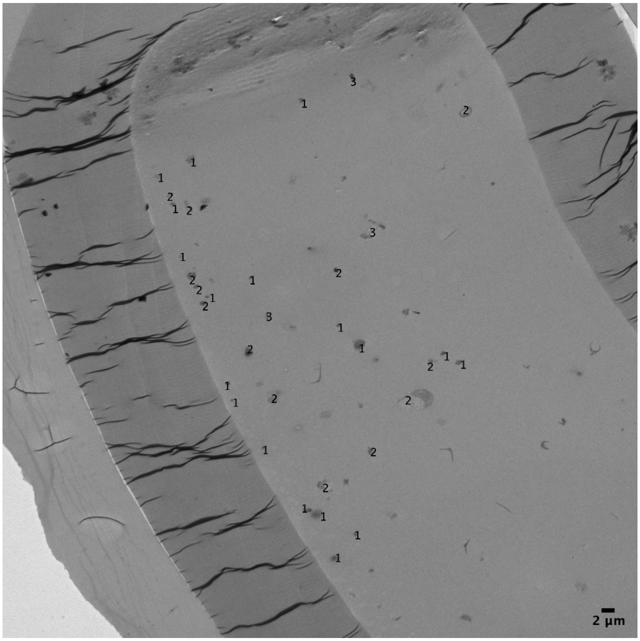

S6g

D.PC overview 3

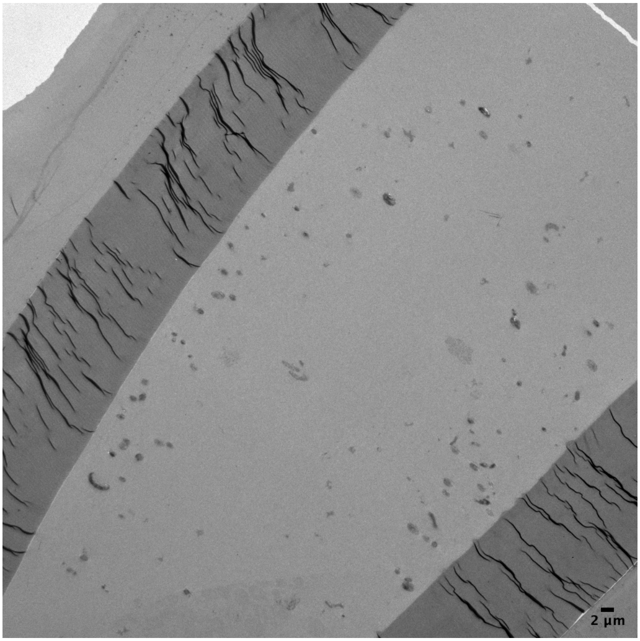

D.PC overview 3 with cell count

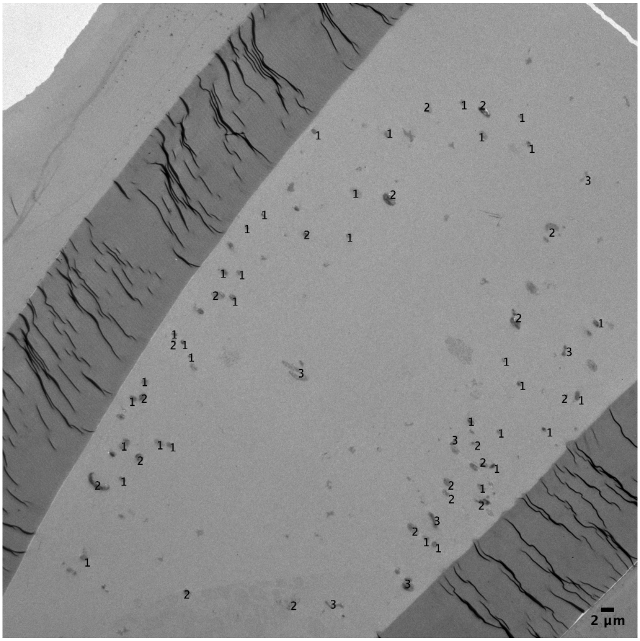

S6h

FBS — Normal | **FBS — Internal Membranes** | **FBS — Intercellular Tubules**

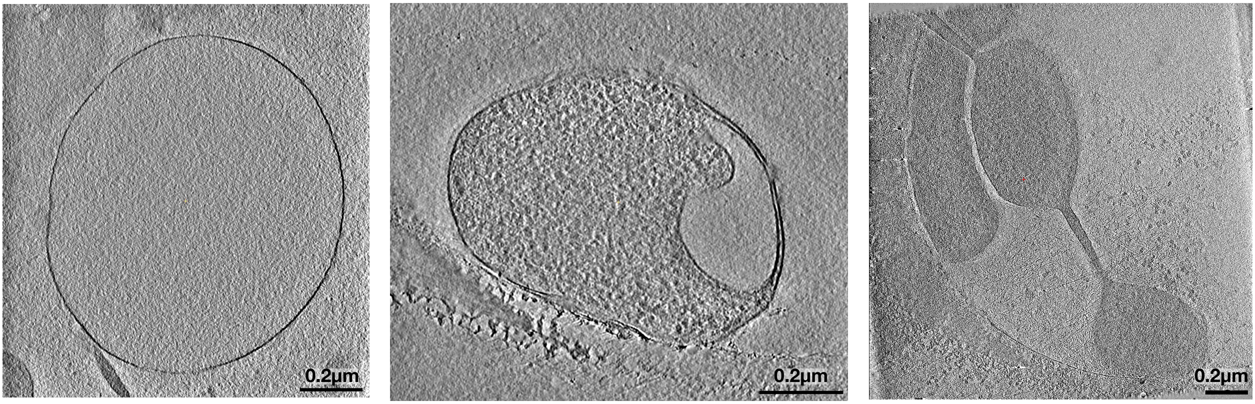

S6i

POPC — Internal Membranes

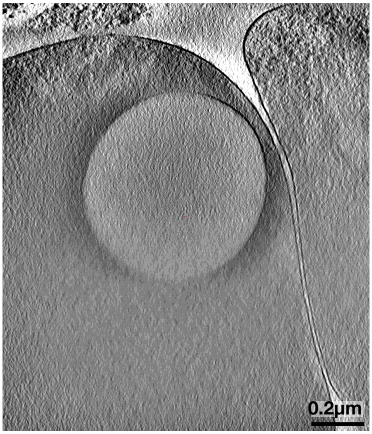

S6j

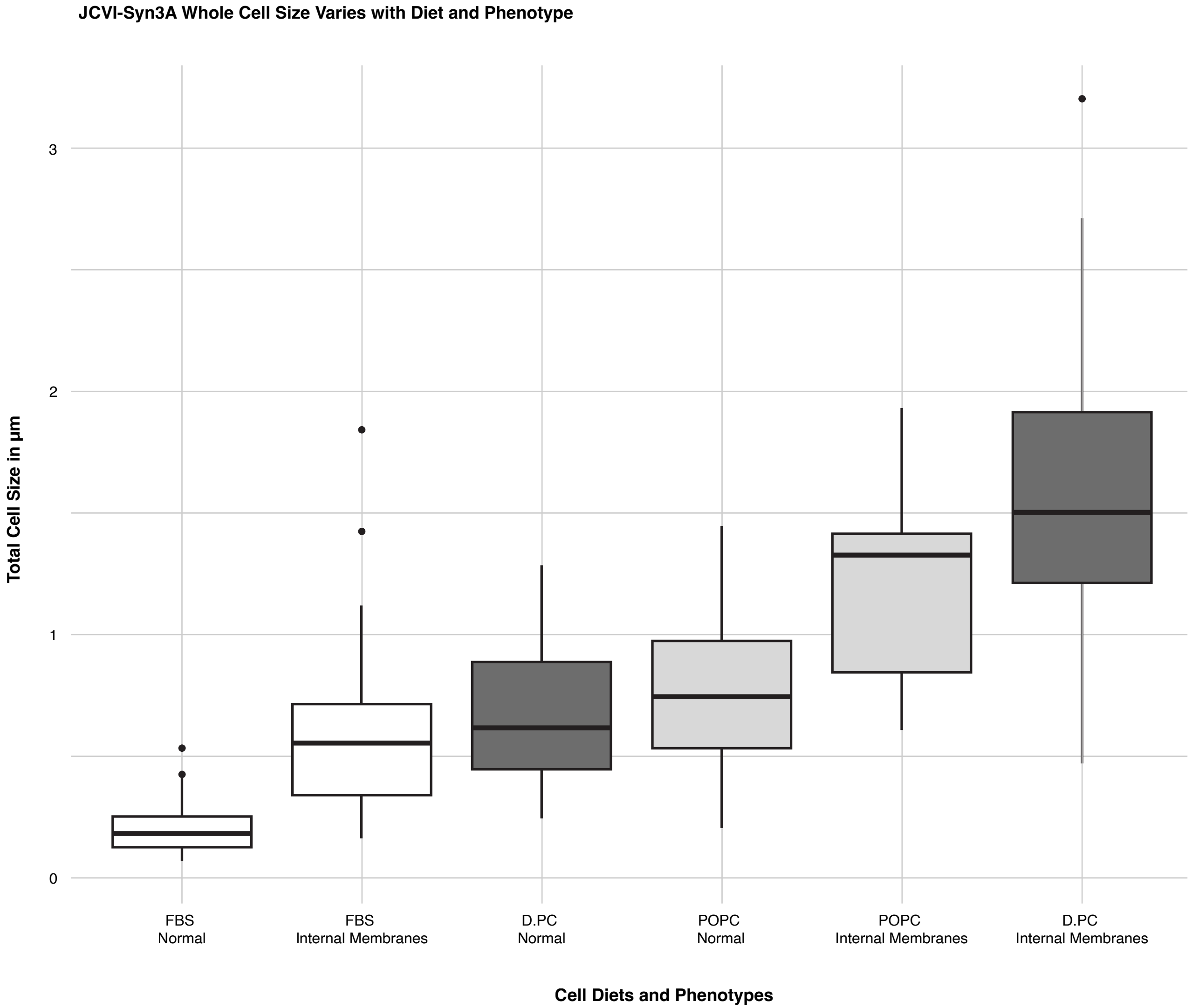

Figure S6: TEM images of JCVI-Syn3A cells display three distinct phenotypes

a-g TEM overview images of capillaries for JCVI-Syn3A cells on FBS, POPC, and Diether PC diets show a distribution of cells displaying Normal morphology, Internal Membranes, and Intercellular Tubules. For each diet, cells were manually counted to find the number displaying each phenotype. **h** 2D slices of Cryo EM tomograms of FBS cells, displaying cells with the Normal morphology, Internal Membranes, and Intercellular Tubules as well, argue against the possibility that these phenotypes are a fixation artifact. **i** 2D slice of a Cryo EM tomogram of a POPC cell with Internal Membranes. The images of “FBS Internal Membranes” and “POPC – Internal Membranes” in this figure, and the Diether PC cells with Internal Membranes in main Figure 4d, collectively show Cryo EM tomograms of all three diets with internal membranes. **j** Average cell diameters vary as a function of both diet and phenotype; with Internal Membrane phenotypes displaying both a much larger cell diameter and a larger range of diameters than Normal phenotype cells of the same diet.

S7a

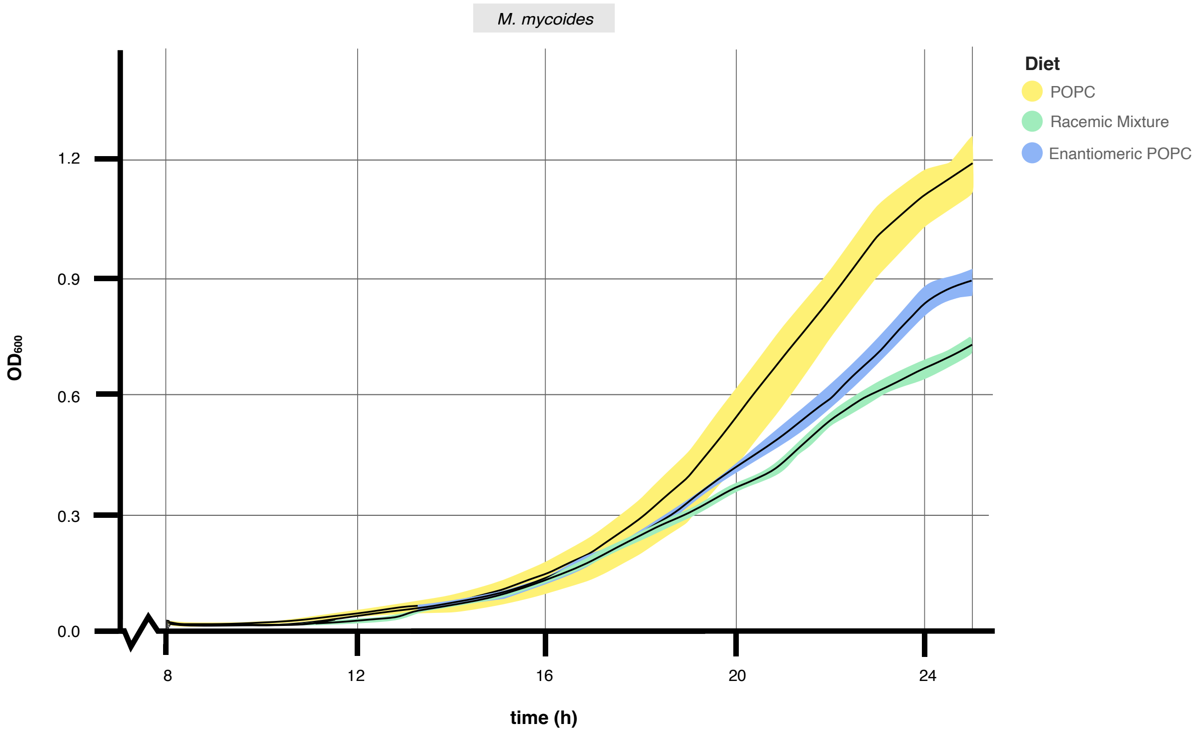

S7b

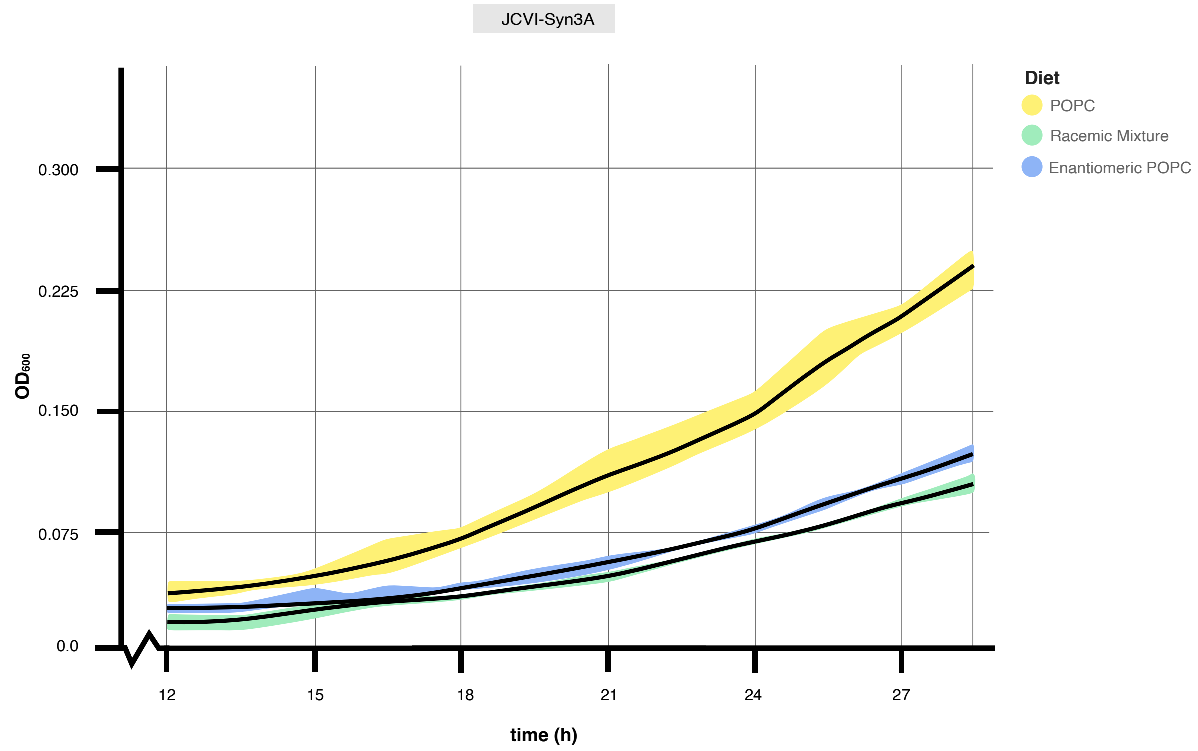

Figure S7: Growth of *M. mycoides* and JCVI-Syn3A manually measured by OD_{600}

a Growth curves for *M. mycoides* on three enantiomeric POPC diets obtained by manual measurements of OD_{600}. **b** Growth curves for JCVI-Syn3A on three enantiomeric POPC diets obtained by manual measurements of OD_{600}. For both cell types, diets with the enantiomer grow slower than the POPC diet. Complementary phenol red A_{562} growth curves for these diets are shown in Figure S4c.
