# Supplementary material for: A tuneable minimal cell membrane reveals that two lipid species suffice for life
Source: Nat Commun. 2024 Nov 8;15:9679. doi: 10.1038/s41467-024-53975-y (PMC11549477; doi:10.1038/s41467-024-53975-y)
Supplement: Supplementary file 2 — Description of Additional Supplementary Files [file 41467_2024_53975_MOESM2_ESM.pdf]

Description of Additional Supplementary Files

Supplementary Data 1. Names and concentrations of lipids used in the “defined diets” described in this experiment.

Supplementary Data 2. Defined diets fed to *M. mycoides* and JCVI-Syn3A, as well as the number of surviving replicates on each diet and their mean growth rate.

Supplementary Data 3. Lipidomic mass-spec data shown in the paper on a replicate level.
